# And if the discovery of new drugs for the treatment of brain diseases depends on Asian countries?

**DOI:** 10.3402/jmahp.v2.24379

**Published:** 2014-06-11

**Authors:** Jean-Louis Kraus

**Affiliations:** Biomolecular Chemistry Team, IBDM (Institut de Biologie du Développement de Marseille Luminy), UMR 7288 CNRS-AMU (Aix Marseille University), Marseille, France

**Keywords:** brain diseases, drug discovery, Asian countries, precautionary principle

## Abstract

At the present time, developed countries are making a huge financial effort to support neuroscience research programs, particularly in the fields of advanced research and treatment of brain diseases and mental disorders. A part of this financial support is devoted to drug discovery programs. The purpose of this communication is to focus on the different parameters (economic, social, and scientific) allowing for the prominent belief that the discovery of new efficient drugs to treat brain disease to an increasing extent is likely to emanate from the Asian countries. A special focus on drug research and discovery in France reveals that, due to the current social context, the lack of small pharmaceutical ventures, the Mediator drug scandal, and the economic situation, the potential for discovering and developing new drugs is dramatically declining.

France was engaged in a plan called *plan Alzheimer 2009–2012* (1) with 44 recommendations promoting the creation of an infrastructure and accountability mechanism to make allowances for the building of dementia-capable programs for the growing number of people with the disease. One of these recommendations was ‘to support research, to inform on the disease, and to organize an epidemiologic follow-up’. An extension of this plan will soon be presented (2) with the aim to improve research and access to new, innovative drugs.

Taking into consideration the following parameters, namely

- the economic international research context (3),
- the specificity of the brain-related pathologies and the neurodegenerative diseases (Alzheimer's Diseases, AD),
- the ‘Mediator case’ that left Servier, the French drug company, as well as the regulators, the industry, and the medical community reeling (4). French regulators withdrew Servier's Mediator (benfluorex) (5) from the market in late 2009. This drug was used off-label for the treatment of obesity and caused between 500 and 2,000 deaths during its tumultuous 33 years on the market,
- the drastic standards foisted by the international authorities upon the pharmaceutical industry for obtaining marketing approvals and conducting clinical trials,

one has to ask the question ‘what is the future of brain disease research and, more specifically, how can we advance toward achieving the discovery of an AD treatment?’

## The precautionary principle applied to drug discovery is a double-edged weapon

At the end of the preclinical phase of research, the safety and the efficacy of a new-found therapeutic substance have to be tested and proved in humans. Under the control of the European Medicines Agency (EMA) (6), and on the grounds of the precautionary principle, EU life science lawyers regularly advise pharmaceutical clients on good clinical practice (GCP), marketing authorizations, importation and manufacture of medicinal products and ingredients, good manufacturing practice (GMP), drug safety, marketing and advertising of medicinal products, pricing and reimbursement, labeling, environmental issues, counterfeit products, and compassionate use. Originally outset in the environmental field during the 1970s, the precautionary principle has gained political profile in the 1980s and 1990s and has attracted the attention of many players involved in matters of environmental protection. Despite its wide resonance, there is no unified definition of the principle. Quite simply, it is usually given to understand that the lack of scientific certainty must not be used as an argument to ignore or postpone preventive or remedial action – as has happened repeatedly in the past – when there is no other good reason to do so. In other words, the precautionary principle sights to protect human health, and to define whether or not a new drug is effective and well-tolerated, as well as to determine its benefit–risk ratio (7).

No one doubts the need for good regulation to secure drug safety. It is likely that, if the precautionary principle was strictly observed, the Mediator (benfluorex) would never have obtained a marketing approval from the French Health Technology Assessment agency. Indeed, while this drug was originally licensed in France for use in the treatment of diabetes, it was mainly prescribed as an anti-obesity drug, and ultimately caused valvular damage and pulmonary arterial hypertension in many patients. Following this incident, the French government should have overhauled its drug safety agency since it is mandated by the government to monitor the drugs side effects and to withdraw unsafe substances from the market, which in this case it failed to do so. Or else the drug should, at the very least, have been quickly withdrawn from the market during the safety phase.

Numerous drugs approved by the Food and Drug Administration (FDA) or having obtained AMM (Autorisation de Mise sur le Marché) marketing authorization in France have been withdrawn from the market due to their poor benefit–risk ratio or during the post-marketing surveillance period because of their alarming side effects (8). The precautionary principle slows the momentum and hinders progress since the drug surveillance systems within countries of the European Economic Community are sufficient to ensure that unsafe drugs are quickly removed from the market. Furthermore, as we tackle the issue of the precautionary principle, one should keep in mind the case of the first, extremely toxic anti-HIV drug (polyoxometalate HPA23) (9) which had been prescribed on a fast track approval mode to the first HIV patients (such as the movie star Rock Hudson). When the first anti-AIDS drug, HPA23, was experimented on humans, it did not meet the standards levied by the precautionary principle; the drug was the forerunner to the discovery of new, efficient anti-AIDS drugs. It might be recalled that, at the beginning, nobody wanted to venture into the field of anti-AIDS drug, because no one thought it was ever going to happen. All these suggest that the precautionary principle (PP) is necessary, because the drug surveillance systems can fail; but PP can also be an obstacle to the discovery of innovative medicines. It should be highlighted that the organization established by the surveillance systems is more favorable to the development of new drugs because products with potential therapeutic benefits, but uncertain safety profile, can reach more patients quickly and hence can accelerate the development of new safer drugs in a given therapeutic field.

## Specific impact of the precautionary principle on drug discovery aimed at curing neurodegenerative or brain disease

Beyond the precautionary principle, there is an increasing general distrust with respect to drugs. Today, everybody wants to have access to safe drugs without side effects. Unfortunately, the drugs acting on the nervous system axiomatically engender side effects, which can disqualify the product, even if it is effective on a small number of patients. In the long run, all psychiatric drugs tend to disrupt the normal processes of feeling and thinking, making the individual less capable of effectively dealing with their personal problems and life challenges. They degenerate the individual's overall mental condition and produce potentially irreversible harm to the brain (10). Most of the current drugs that act on the nervous system, launched in the past 50 years, at a time when only very few things were known about the functioning of the human brain, would not come out today because of their side effects. Many drugs as useful as nerve sedatives and neuroleptics, such as phenothiazine (11), or drugs such as aspirin, digitalin, and morphine would probably not receive approval to day from the FDA or the EMA, if the value of these drugs had to be assessed on the premise of the current regulatory standards concerning safety.

Why is it becoming increasingly harder to make new drugs? The Big Pharma is no longer governed by physicians, biologists, or medicinal chemists but by financial managers, who appreciably decrease the aerofoil of the neurological research program on which they had wasted a lot of money during the past few years (12). They prefer to invest in research programs in which they are certain to recover substantial benefit; between Viagra and Alzheimer’ disease, their choice is quickly made.

## The case of AD research

AD will affect more than 20 million people in France in 20 years (13). Unfortunately today drugs for the treatment of AD are not available, but if they were, to whom would they be prescribed?

- To patients in the early stages of the disease to circumvent further development?
- To patients already at an advanced stage of the disease?

These questions are difficult to answer. Once a patient has been clearly diagnosed with AD, the prospect of restoring their normal neuroplasticity through the use of drugs becomes uncertain for a majority of the neurobiologists’ community. Confronted with this cruel reality, AD research programs should focus on the following main topics:

- Tests (blood, genomic) to identify patients at risk.
- Search for early AD detection methods. If the disease is diagnosed sufficiently early, it might be possible to stimulate a spontaneous repair of the nervous system. We know that brain repair occurs in some cases of stroke or brain injuries.
- Continuation of fundamental research to establish AD causes.
- Search for new drugs aiming to slow down the progression of the disease, or to allow for the social rehabilitation of the patients.

In Western countries with universal health coverage, especially in France, a drug is regarded as profitable for pharmaceutical manufacturers only if it is reimbursed by social security.

Consequently, the search for new AD drugs could turn out to be more profitable for the pharmaceutical industry if conducted in countries without universal health coverage or in countries rated as flexible with regard to the application of the precautionary principle and the norms for clinical trials.

These countries represent two thirds of the planet, and many of them are located in Asia. Countries such as India and, *a fortiori*, China could accommodate the research and the discovery of new AD drugs. During the past few years, the rising cost and the decreasing drug approval rate have coerced the multinational pharmaceutical companies (MNCs) to relocate at least a part of their clinical trials to these Asian countries.

Besides the lower costs, they seek to procure their own share of the market within these populated countries. Obstacles such as intellectual property protection, regulatory policy, trials quality, and approval have been a major concern for the MNCs conducting clinical trials in these countries. Above all else, these trials needed to be revamped and lined up with the international standards (14, 15). Chinese HTA, with an open and positive attitude, have put efforts in amending their regulations and policies to enable more efficient and time-effective approval means, as well as transparent processes and internationally recognized guidelines by means of intensive cooperation particularly with the US FDA (16).

In India, clinical trials declined drastically after the government embroiled the regulations for clinical trials and put the onus of the participants safety on the firms conducting them. It has been assumed that 2,262 people died during the past 5 years, leading to a public outcry and a Supreme Court order in favor of stricter norms for holding drug trials (17). Nevertheless, India's clinical research market was projected to be more than 1 billion USD by 2016 driven by a large and easy-to-access population with much lower cost than in the developed world (17).

In this context, the hope to discover new drugs that would be approved for neurodegenerative diseases will probably come from Asian countries such as China, where the population age structure, coupled with the one child policy, has encouraged the government to gradually open FIH (First in Human) trials for international companies. Asian countries will also have the opportunity to participate in the development of new drugs by way of promoting and breeding joint ventures with MNCs.

## Why French research programs for drug discovery are on the decline, particularly for drugs devoted to the treatment of brain disease?

The first reason is that the Mediator disaster in France along with the media speculation on and about a recent publication listing useful, useless, or dangerous drugs, and which can prompt a decision to emend the drug reimbursement policies (18), have occasioned an increased distrust toward the drug industry, with a detrimental impact on both public and private research. The second reason is that the academic medicinal chemistry researchers, involved in a brain disease drug discovery program, have actually succeeded in identifying active drugs in vitro but have failed to obtain financial support from the private pharmaceutical companies for the clinical development of the drug, because of the high cost of clinical trials, the long intervening time before the marketing of the product, the drastic norms to conduct the trials and, in the specific case of AD drugs, the low odds of discovering a drug that both meets those drastic standards and yet yields a significant benefit–risk ratio. Given the huge number of potential customers, investing in neurodegenerative diseases may seem like an incontrovertible strategy, yet most of the clinical trials for AD drugs have failed so far, and in the past years different companies have cut down their investment in Central Nervous System, Research and Development (CNS R&D) (19).

The current trend of thought about stalling AD reckons that music stimulation, intellectual and mental activity may have a greater positive impact than drugs. In this perspective, AD research programs are more and more oriented toward the discovery of drugs that help or support the rehabilitation of AD patients. The goal is to assist the neurodegenerative disease patients to grow increasingly autonomous; otherwise, the French social security system may collapse in the near future.

We would like to conclude this short report on brain drug discovery in France with a significant example that epitomizes the kind of difficulties encountered by a French start-up company founded by a group of academic researchers to develop new drugs in the field of brain cancer. Glioblastoma (GBM) is a rare, classified disease (20), and the single most aggressive malignant primary brain tumor in humans. The only clinically approved drug used worldwide is temozolomide (TMZ) often coupled with radiotherapy, as this drug seems to proceed by sensitizing the tumor cells to the radiation. The promoters of the company have discovered a promising family of compounds equally active to TMZ on GBM xenografted mice, with an original action mechanism and with a demonstrated synergistic effect with TMZ. The new start-up company could not afford the financial cost for phase 1 clinical trials. As they petitioned for partnership with a pharmaceutical company or else funding from a venture capital firm, the recurrent answer was:

- GBM is a rare disease with a narrow market size; your project is too vernal and too risky; come back as soon as you have completed phase 1 or 2 of the clinical trials.

A way forward for this start-up company to collect additional funds in order to initiate the first trial of these new anti-glioblastoma drugs on humans could be to arrange partnerships with Asian pharmaceutical companies.

We have shown that neurological research in France is not attractive enough for investors and, therefore, France is at risk of losing a part of its research volume to Asian countries. From a therapeutic point of view, this movement of research in the direction of the Asian countries may have a positive impact on human health by accelerating the research and discovery of brain medicine; nevertheless, it also has a negative economic impact on the countries that lose a part of their potential for drug discovery.
